# Optimization and Characterization of the F-LSR Manufacturing Process Using Quaternary Ammonium Silanolate as an Initiator for Synthesizing Fluorosilicone

**DOI:** 10.3390/polym14245502

**Published:** 2022-12-15

**Authors:** Jae Il So, Chung Soo Lee, Ji Young Jung, Jaewon Lee, Jin Kyu Choi, Sang Eun Shim, Yingjie Qian

**Affiliations:** 1 Department of Chemistry and Chemical Engineering, Education and Research Center for Smart Energy and Materials, Inha University, Incheon 22212, Republic of Korea; 2 Grace Continental Korea Co, 101-303, Bucheon Techno-Park, 364, Samjeong-Dong, Ojeong-Gu, Bucheon-Si 14501, Republic of Korea

**Keywords:** fluorosilicone, anionic-ring-opening-polymerization, liquid silicone rubber, cold resistance

## Abstract

Due to the growing demand for versatile hybrid materials that can withstand harsh conditions (below −40 °C), fluorosilicone copolymers are becoming promising materials that can overcome the limited operating temperature of conventional rubber. In order to synthesize a fluorosilicone copolymer, a potent initiator capable of simultaneously initiating various siloxane monomers in anionic ring-opening polymerization (AROP) is required. In this study, tetramethyl ammonium silanolate (TMAS), a quaternary ammonium (QA) anion, was employed as an initiator for AROP, thereby fluoro-methyl-vinyl-silicone (FVMQ) and fluoro-hydrido-methyl-silicone (FHMQ) were successfully synthesized under optimized conditions. FT-IR, NMR, and GPC analyses confirmed that the chain length and functional group content of FVMQ and FHMQ are controlled by changing the ratio of the components. Moreover, fluorine-involved liquid silicone rubber (F-LSR) was prepared with FVMQ as the main chain and FHMQ as a crosslinker. The tensile strength, elongation, and hardness of each F-LSR sample were measured. Finally, it was confirmed through TGA, DSC, TR-test, and embrittlement testing that elastic retention at low temperatures improved even though the heat resistance slightly decreased as the trifluoropropyl group increased in F-LSR. We anticipate that the optimization of fluorosilicone synthesis initiated by QA and the comprehensive characterization of F-LSRs with different fluorine content and chain lengths will be pivotal to academia and industry.

## 1. Introduction

The spotlight on organic–inorganic hybrid materials in modern industry and academia is based on their characteristics [1]. Silicone rubber, a representative hybrid material, has special features originating from its unique molecular structure [2]. Silicone rubber, with the general formula (R_2_SiO)_n_, is composed of a robust inorganic backbone and organic functional groups with various properties. Polydimethylsiloxane (PDMS), which contains a large number of methyl groups, is widely used in various fields owing to its superior flexibility, heat resistance, cold resistance, biocompatibility, and air permeability, compared with conventional polymers [2,3]. The growing and exact demand for realizing the stable operation of instruments in the space, defense, and automobile industries require silicone rubber to withstand harsher conditions than the existing PDMS. In order to meet these goals, silicone polymers have been diversely functionalized, and fluorosilicone is one of the most reliable candidates.

Fluorosilicone, which contains siloxane segments and fluorinated groups, has substantially different properties than PDMS owing to the presence of fluoroalkyl groups [4]. Among fluorosilicones, the most widely studied material is poly[(3,3,3-trifluoropropyl)methylsiloxane] (PTFPMS). PTFPMS has various properties, such as waterproofing, resistance to oil, fuel, and solvents, low-temperature resistance, and excellent film-forming properties derived from the trifluoropropyl groups and siloxane backbone [5,6,7,8,9,10]. This multifunctional advantage of PTFPMS can be employed as a protective coating, antifoaming agent, eye-curing agent, and adhesive. Furthermore, fluorosilicone elastomers are widely used as sealing materials for gasoline, fuel, and aircraft because of their ability to seal at low temperatures and reduce the possibility of fuel penetration. However, PTFPMS has several drawbacks regarding high-temperature stability and mechanical properties compared to conventional PDMS because the trifluoropropyl groups in PTFPMS are more susceptible to homolytic scission at high temperatures [11]. In order to overcome these limitations of fluorosilicone, it is essential to properly copolymerize it with a siloxane that has a methyl or vinyl group when synthesizing fluorosilicone [6].

In general, fluorosilicone is synthesized through the ring-opening polymerization (ROP) of 1,3,5-trimethyl-1,3,5-tris(3,3,3-trifluoropropyl)cyclotrisiloxane (F-D_3_). Compared to the hydrolysis-condensation of fluorosilanes, synthesizing fluorosilicone through ROP has advantages, such as facile reactions, inexpensive raw materials, and a high molecular weight [12]. The mechanism of ROP consists of the Si–O bond of cyclosiloxane being cleaved by an anion or a cation, which occurs because of the ring strain of the Si–O bond of cyclosiloxane resulting from the great electronegative difference between Si and O [4]. The reaction can be initiated by alkaline or acid substances, which are referred to as anionic ring-opening polymerization (AROP) or cationic ring-opening polymerization (CROP), respectively. In particular, AROP is the most famous method for synthesizing fluorosilicone because of the ease of controlling its viscosity and the clarity of its mechanism [13]. In the AROP process, the initiator is a crucial factor in determining the reaction rate. The polymerization rate, yield, and molecular weight of the products can be controlled depending on the type and amount of initiator. The most widely used initiator for fluorosilicone synthesis is potassium hydroxide [5,14,15,16]; additionally, sodium hydroxide [17,18] and lithium hydroxide [19] have also been employed. Moreover, the quaternary ammonium base (QA), compared to conventional bases, is the major initiator.

QA is a very useful initiator for fluorosilicone synthesis, reducing the reaction temperature and number of processes. First, the reactivity of QA, except ammonium hydroxide, is exceptionally high because of the bulky size of the counterion [20,21,22]. Tetramethyl ammonium hydroxide (TMAH), a representative material of QA with the molecular formula N(CH_3_)_4_^+^ OH^−^, can initiate AROP with F-D_3_ at 25 °C [4]. When compared with other alkaline initiators (KOH: 70 °C, NaOH: 90 °C, and LiOH: 120 °C), AROP that uses TMAH can react at lower temperatures [4,5,23]. Additionally, diverse monomers with different reactivities must react simultaneously to form silicone copolymers. Therefore, when reacting various cyclosiloxane monomers, a highly reactive initiator is required to readily reach reaction equilibrium. Second, the reaction could be quickly terminated by a simple neutralization process. In most AROPs of cyclosiloxane, catalyst neutralization is significant for the success of the overall process, as basic catalyst residues lead to polymer degradation [4]. In general, after AROP, additional neutralization using substances such as silyl phosphate, hydrochloric acid, and CO_2_ is required, whereas, in the case of QA, counterions can be simply removed by heating above 130 °C [15,24]. Many studies have been conducted on silicone synthesis using QA [25,26], but few studies have comprehensively evaluated the factors influencing the fluorosilicone copolymerization process with various monomers and their effect on the properties of synthetic products. Since the reaction rates of F-D_3_ and D_4_ are different when producing fluorosilicone, it is necessary to evaluate the effect of the reaction rates on the reaction and optimize the reaction conditions when using QA as an initiator. In addition, it is required to evaluate the influence of the molecular length and fluorine content of the synthesized fluorosilicone on the mechanical and thermal properties of the synthesized fluorosilicone.

In this study, we synthesized fluoro-vinyl-methyl-silicone (FVMQ) as the main chain of fluorosilicone rubber and fluoro-hydride-methyl-silicone (FHMQ) as a crosslinker of fluorosilicone rubber, using TMAS as the initiator. In addition, liquid-fluorosilicone rubber (F-LSR) with various viscosities and fluorine content was prepared by hot press molding by adding silica as a filler. Finally, the thermal and mechanical properties of the synthesized F-LSRs with different molecular weights and fluorine content were investigated. In addition to illustrating a reliable method of producing F-LSR by considering the reaction conditions, fluorine content, and molecular weight, we believe that this work will help researchers to understand the thermal and mechanical properties of F-LSR.

## 2. Materials and Methods

### 2.1. Materials

All chemicals and solvents were commercially available and used without further purification. TMAH (25% in water) and methanol were purchased from Alfa Aesar (Haverhill, MA, USA). Octamethylcyclotetrasiloxane (D_4_, >99%), tetramethyltetravinylcyclotetrasiloxane (Vi-D_4_, >99%), tetramethylcyclotetrasiloxane (H-D_4_, >99%), hexamethyldisiloxane (MM, >99%), and divinyltetramethyldisiloxane (ViVi, >99%) were purchased from Dami Polychem (Iksan, Republic of Korea). 1,3,5-trimethyl-1,3,5-tris(3,3,3-trifluoropropyl)cyclotrisiloxane (F-D_3_, >99%) was purchased from HRS Corporation (Seoul, Republic of Korea). Fumed silica was purchased from Grace Continental Korea (Bucheon, Republic of Korea) to prepare the liquid silicone.

### 2.2. Synthesis of TMAS

TMAS was synthesized by the reaction of TMAH (0.3 mol) and D_4_ (0.3 mol) in a 500 mL four-neck flask equipped with a mechanical stirrer. The reactant was stirred at 200 rpm under an argon atmosphere at 80 °C for 48 h. The product was obtained as a well-dissolved solution with a 90% yield (Figure S1).

### 2.3. Synthesis of FVMQ

All FVMQs were synthesized in a 1 L four-neck flask with a mechanical stirrer and reflux condenser. Before initiating polymerization, D_4,_ F-D_3_, and Vi-D_4_ were charged into a flask under an argon atmosphere at 90 °C for 1 h to maintain the initiating temperature. Thereafter, the initiator TMAS and end-blocker ViVi were added to the solution, and the solution was stirred at 300 rpm under an argon atmosphere for 0.5–5 h. After the reaction, the product was heated to 150 °C and maintained for 24 h under vacuum conditions to remove the unreacted reactant and initiator. Finally, all products were purified with methanol to eliminate the cyclic monomers. The purified FVMQs were transparent liquids with high viscosities, with yields of 50–95% (Figure S1).

### 2.4. Synthesis of FHMQ

All FHMQs were synthesized in a 1 L four-neck flask with a mechanical stirrer and reflux condenser. First, D_4_ and F-D_3_ were charged into a flask under an argon atmosphere at 90 °C for 1 h to maintain the initiating temperature. The reaction was initiated by the addition of TMAS into the reactor at 300 rpm and 90 °C. After initiating the reaction, a certain amount of H-D_4_ was added dropwise to this solution within 0.5 h under vigorous stirring using a 250 mL dropping funnel. Thereafter, the end-blocker of MM was added to the reactor, and the mixture was stirred at 90 °C for 1–4 h. After the reaction, the products were heated to 150 °C for 24 h under vacuum conditions to remove unreacted reactant and initiator. Finally, all products were purified with methanol to eliminate the cyclic monomers. The purified FHMQs were transparent liquids with a lower viscosity than FVMQ, giving yields of 75–83% (Figure S1).

### 2.5. Preparation of F-LSR

To prepare the F-LSR, the synthesized product was divided using a two-part kit and mixed. Kit A was composed of 100 weight parts of FVMQ, 10 weight parts of fumed silica, and 0.1 weight parts of Karstadt’s. Kit B was composed of 100 weight parts of FVMQ, 10 weight parts of fumed silica, and 10 weight parts of FHMQ. Each kit was placed in a vacuum oven at 100 °C for 2 h to remove residual moisture and air bubbles from the mixture. Subsequently, the exact amounts of kit A and kit B were mixed using a hand-mixer at 300 rpm, and the mixture was directly injected into the molding cavity for hot-press molding. All F-LSRs were compression molded at 160 °C for 5 min and post-cured at 200 °C for 5 h in an oven (Figure S1).

### 2.6. Characterization

Attenuated total reflection Fourier transform infrared spectroscopy (ATR-FTIR) spectra were obtained using a Spectrum 2 spectrometer (PerkinElmer, Waltham, MA, USA) with a resolution of 1 cm^−1^ by acquiring 16 scans. The analysis was performed within a frequency range of 4000–400 cm^−1^.

^1^H-NMR at 400 MHz (Bruker Advance III spectrometer (Bruker, Karlsruhe, Germany)) and ^29^Si-NMR at 100 MHz (Avance III-500 (Bruker, Karlsruhe, Germany)) were performed to elucidate the detailed chemical structure and calculate the ratio of each functional group in the fluorosilicone. All samples were prepared in 0.6 mL of CDCl_3_, and the NMR spectra were recorded.

Viscosity was measured using the DV1 viscometer (Brookfield Engineering, Middleboro, MA, USA). All samples were measured in equal volumes of 300 mL at 25 °C. The spindle type and rotation speed were adjusted such that the torque value was between 50 and 60% when measuring the viscosity of all samples.

The weight-average molecular weight and dispersity of FVMQ were measured by gel permeation chromatography (GPC) using an isocratic high-pressure pump (S9430 (Schambeck SFD, Bad Honnef, Germany)) and a refractive index detector (S2020 (Schambeck SFD, Bad Honnef, Germany)). A sample of 50 mg was dissolved in the mobile phase (tetrahydrofuran), and the toluene solution was allowed to flow at a rate of 1 mL/min at 40 °C.

Tensile tests of the F-LSRs were carried out according to the ASTM D-412 method using a universal testing machine. All test specimens were dumbbell-type 1 at room temperature with a crosshead speed of 500 mm/min. The hardness of the F-LSR was analyzed according to ASTM D2240-15el using an ASKER Durometer type A (KOBUNSHI KEIKI CO, Kyoto, Japan).

Thermogravimetric analysis (TGA) was conducted using TGA 4000 (PerkinElmer, Waltham, MA, USA), in which the samples were heated from 40 to 750 °C at a heating rate of 10 °C/min under a nitrogen atmosphere. The weight loss rate curves of all samples were obtained by differentiating the TGA thermograms.

Differential scanning calorimetry (DSC, DSC 8000 (Perkin Elmer, Waltham, MA, USA)) was performed to investigate the thermal properties at low temperatures. DSC measurements of all samples using 5–10 mg was performed from −130 to 30 °C at heating and cooling rates of 10 °C/min.

The low-temperature properties of F-LSR were analyzed using combined low-temperature testers (ET05, Elastocon, Springfield, IL, USA). Temperature-retraction test (TR-test) of F-LSR was conducted in accordance with the ASTM D1329-16 standard, and the brittleness temperature of F-LSR was obtained by a test method in accordance with ASTM D746-20 (Figure S2).

## 3. Results and Discussion

### 3.1. Synthesis of TMAS

**Figure 1:**
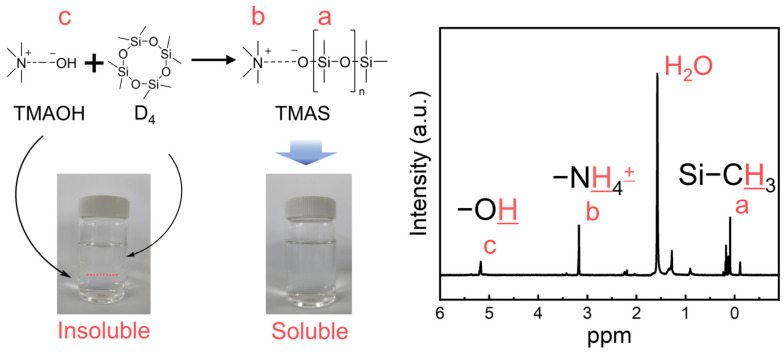
Synthetic pathways and characterization of functional groups from ^1^H-NMR spectrum of TMAS.

TMAS was prepared using TMAH and D_4_ prior to the synthesis of FVMQ and FVMQ. TMAH shows strong initiating performance in the synthesis of silicone with a solvent. However, because this reaction was a bulk polymerization without a solvent, the initiating performance of TMAH might be decreased because of the immiscibility of TMAH and the siloxane monomer. Therefore, the modification of TMAH to TMAS is required. The differences in the solubility of the initiator before and after the reaction are shown in Figure 1. Before the reaction, TMAH and D_4_ were not dissolved with each other, but it was confirmed that the TMAS after the reaction became a clear single phase. NMR data confirmed that the peaks of Si–CH_3_, N–CH_3_, and OH at 0, 3, and 5.5 ppm, respectively, coincided with the theoretical value.

### 3.2. Synthesis of FVMQ

**Figure 2:**
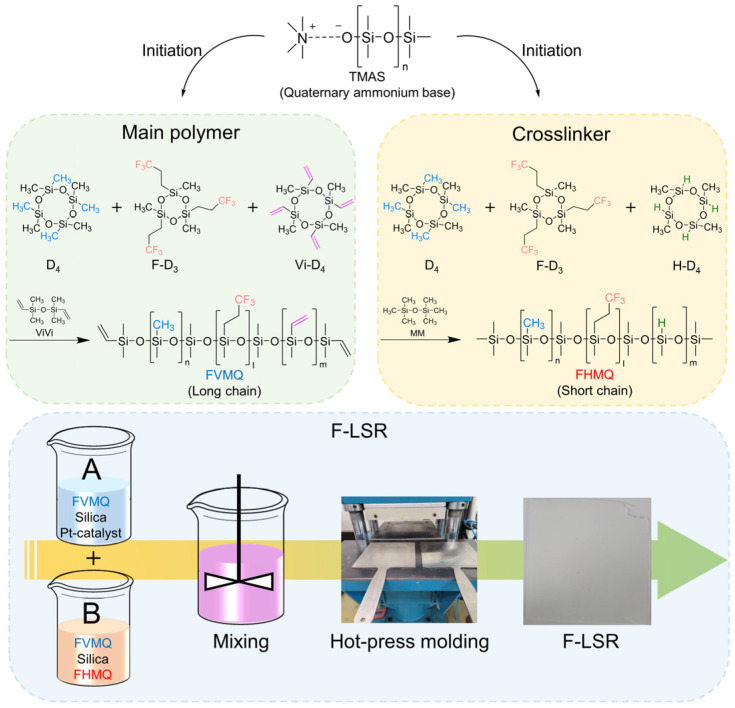
Schematic of all processes for synthesizing fluorosilicone and manufacturing liquid silicone rubber.

FVMQ was prepared by AROP using TMAS as the initiator; the synthetic procedure is illustrated in Figure 2. The D_4_, F-D_3_, and Vi-D_4_ monomers were used as precursors for the addition of the methyl, trifluoropropyl, and vinyl groups, respectively, in the chain. Finally, an end-blocker for ViVi was added to limit the length of the polymer chain and to determine the functional groups at the end of the chain. All the feed ratios of the synthesized FVMQ are shown in Table 1.

#### 3.2.1. Optimizing the Amounts of Initiator

**Figure 3:**
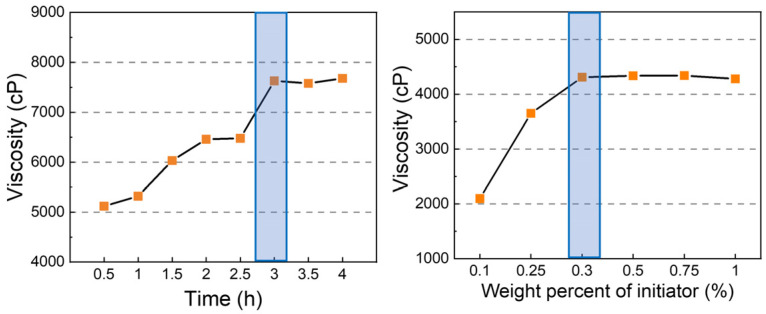
The relationship between the reaction time and viscosity of the FVMQs (left) and the relationship between the weight percentage of the initiator and viscosity (right).

Before preparing FVMQ, which is the main chain of F-LSR, the reaction conditions were optimized because the properties of the product could be significantly affected by the reaction conditions (Table S1). First, the reaction was carried out by varying the reaction time from 0.5 to 4 h, and the viscosity was measured (Figure 3). The viscosity of the product was 5150 cP at a reaction time of 0.5 h, and the viscosity increased as the reaction time increased to 3 h. The reason for the increase in viscosity over time is that FVMQ did not reach reaction equilibrium because the polymerization rate of D_4_ was significantly lower than that of F-D_3_ [27,28]. The reaction time reached equilibrium after 3 h, and the viscosity was fixed at approximately 7500 cP. Secondly, FVMQ was synthesized by changing the amount of initiator under the same reaction conditions. When the ratio of F-D_3_:D_4_ was 7:3, and the reaction time was 3 h, the viscosity of the product increased to 0.3 wt% of TMAS, and the viscosity was maintained above 0.3 wt% of TMAS. According to these results, the following experiments were carried out for 3 h in the presence of 0.3 wt% of the initiator.

#### 3.2.2. Viscosity and Molecular Weight of FVMQ

**Figure 4:**
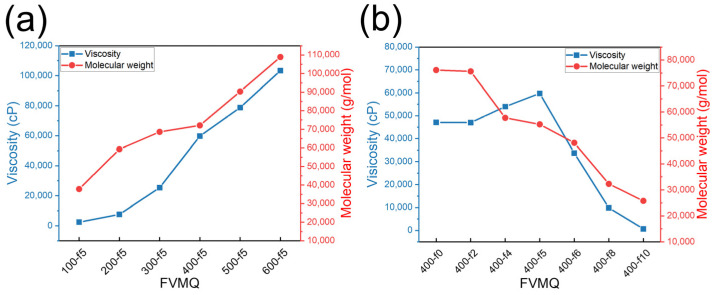
Correlation between the viscosity and molecular weight of FVMQs with (**a**) different chain-lengths and (**b**) different fluorine ratios.

The formula for the reactant ratios of the synthesized FVMQs is listed in Table 2. The viscosity and molecular weight of the FVMQs were analyzed using a viscometer and GPC, respectively. Figure 4 shows the correlation between the molecular weight and viscosity of fluorosilicone. Figure 4a confirms that the greater the difference between the amounts of monomer and end-blocker in AROP, the greater the viscosity and molecular weight. Moreover, when the functional groups of the product are similar, the molecular weight and viscosity are proportional, which is consistent with the general theory [29]. In contrast, the molecular weight decreased as the monomer ratio of F-D_3_ increased, compared to D_4_, as shown in Figure 4b. This AROP reaction is thermodynamically controlled ROP because of the strong QA derived from TMAS [12]. In the thermodynamic reaction, the equilibrium of F-D_3_ between the polymer and cyclosiloxane is biased towards cyclosiloxane. Therefore, the chain length of FVMQ with a higher ratio of trifluoropropyl groups becomes shorter and increases the occurrence of the unexpected side reactions, such as backbiting [15,17,22]. Meanwhile, it was confirmed that viscosity increased slightly and then decreased as the number of trifluoropropyl groups increased. Although the molecular weight decreased slightly from FVMQ-400-f0 to FVMQ-400-f5, the increase in the trifluoropropyl group in the chain contributed to the higher viscosity. The viscosity of PTFPMS is 10 times that of PDMS with the same molecular weight [4]. However, in the FVMQ-400-f6 to FVMQ-400-f10 samples, the molecular weight is small enough to offset the effect of increasing the viscosity by the trifluoropropyl group. Finally, it was confirmed that the viscosity of FVMQ-400-f10 decreased to 670 cP.

#### 3.2.3. Characterization of the Functional Groups of FVMQ

**Figure 5:**
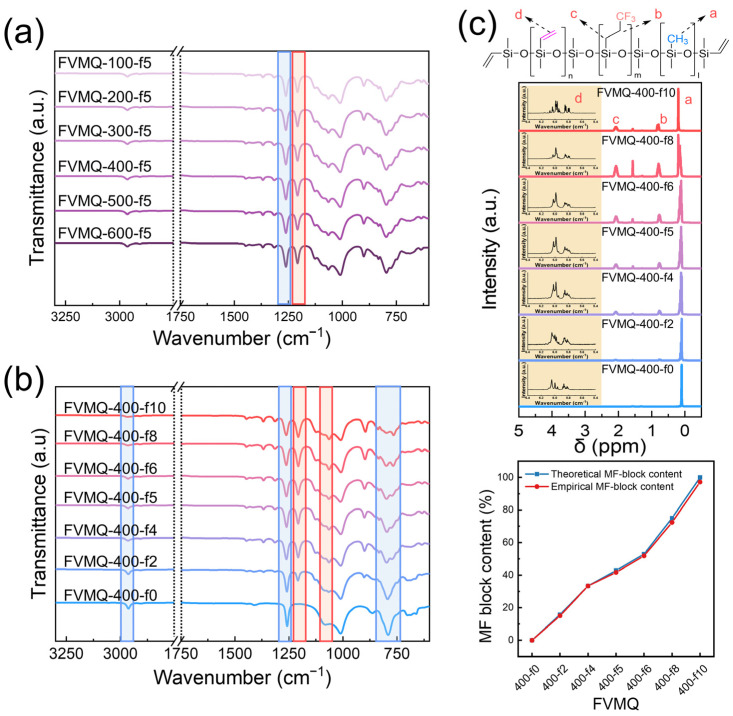
FT-IR spectra of the FVMQs: (**a**) FVMQ-100-f5–FVMQ-600-f5 and (**b**) FVMQ-400-f0−FVMQ-400-f10; (**c**) ^1^H-NMR spectra of FVMQ-400-f0 to FVMQ-400-f10 and a diagram of MF-block content between the theoretical and empirical values.

FT-IR analysis was performed to investigate the functional groups of the FVMQs. Figure 5a,b shows the FT-IR spectra of the FVMQs with different viscosities and ratios of the trifluoropropyl groups. The strong absorption band from 1130 to 1000 cm^−1^ in all spectra was attributed to the –Si–O–Si– asymmetric stretching vibration of the FVMQ backbone [30]. In addition, the absorption band of the–CH bond in –CH_3_ at 2960 cm^−1^ was observed in all spectra of the FVMQs. As shown in Figure 5a, the locations of all absorption bands and peak intensities are not different because the feeding ratio of all samples shown in Figure 5a is identical, except for ViVi [30].

As shown in Figure 5b, when moving from FVMQ-400-f0 to FVMQ-400-f10, the peak ratio of –CF_3_ (1210 cm^−1^) to Si–CH_3_ (1226 cm^−1^) increased. In other words, as the trifluoropropyl group content increased, the peak intensity at 1226 cm^−1^ decreased, and the peak at 1210 cm^−1^ gradually appeared. The peaks at 1315 and 1128 cm^−1^, corresponding to the −CH_2_−CH_2_− and C−H bonds of −CH_2_−, respectively, also increased with the number of trifluoropropyl groups [4]. Meanwhile, the absorption band in the region of 870–750 cm^−1^ originating from −CH_3_ rocking and Si−C stretching changed from a single peak at 2950 cm^−1^ to split peaks between 800 and 775 cm^−1^ when moving from FVMQ-400-f0 to FVMQ-400-f10 [5]. However, the absorption band derived from the vinyl group was not identified in any of the spectra because of the small number of vinyl groups compared with other functional groups.

^1^H- and ^29^Si-NMR spectra were obtained to further investigate the functional groups in FVMQ with different feeding ratios (FVMQ-400-f0 to FVMQ-400-f10) and determine the specific structures of FVMQ. In Figure 5c, a strong peak at approximately 0 ppm is observed for all the FVMQs, which is characteristic of Si–CH_3_ [31]. The peak intensities increase at 0.7 and 2.1 ppm for α-hydrogen and β-hydrogen, respectively, originating from Si-CH_2_CH_2_CF_3_ with increasing F-D_3_ in the feeding ratio [31]. Meanwhile, the spectrum corresponding to the Si–CH=CH_2_ group was confirmed for all samples by expanding the scale of the NMR spectrum to approximately 6 ppm [32]. The quantitative block ratios of dimethyl (DM-block), methyl-fluoro (MF-block), and methyl-vinyl (MV-block) were calculated using the integrated peak areas, as shown in Table 3. The proportion of the functional groups (of the DM-block to the MF-block) calculated using ^1^H-NMR was almost identical to the theoretical proportion, where the errors were less than 2% for all FVMQs. Furthermore, the DM-block of all FVMQs was calculated to be 2–2.5 mol%, which is sufficient for crosslinking with FHMQ. Meanwhile, the ^29^Si-NMR spectra showed that the FVMQs of the copolymerization with F-D_3_ and D_4_ were random copolymers (Figure S3) [33]. Under a TMAS catalysis, the AROP of FVMQ is so fast that the reaction equilibrium is quickly reached, which means that chain distribution occurs frequently.

### 3.3. Synthesis and Characterization of FHMQ

FHMQ was synthesized using a procedure similar to that used for FVMQ synthesis, except for subtle differences. The monomers D_4_ and F-D_3_ were used as precursors for introducing the methyl and trifluoropropyl groups, respectively, and H-D_4_ was employed to insert the hydrido groups instead of Vi-D_4_. In FHMQ, the vinyl group must be avoided because of the possibility of self-crosslinking. Therefore, MM was used instead of Vi as an end-blocker in the FHMQ synthesis. First, the reaction was carried out using F-D_3_ and D_4_. H-D_4_ and an end-blocker were then added dropwise to the reactor over 30 min using a dropping funnel. As the reactivity of H-D_4_ was too high under the above conditions, the amount of TMAS was reduced from 0.3 to 0.1 wt% for decreasing reactivity. All FHMQs were synthesized via AROP with different reaction periods ranging from 1.5 to 5.5 h, named FHMQ-1.5h to FHMQ-5.5h, respectively (Table S2).

**Figure 6:**
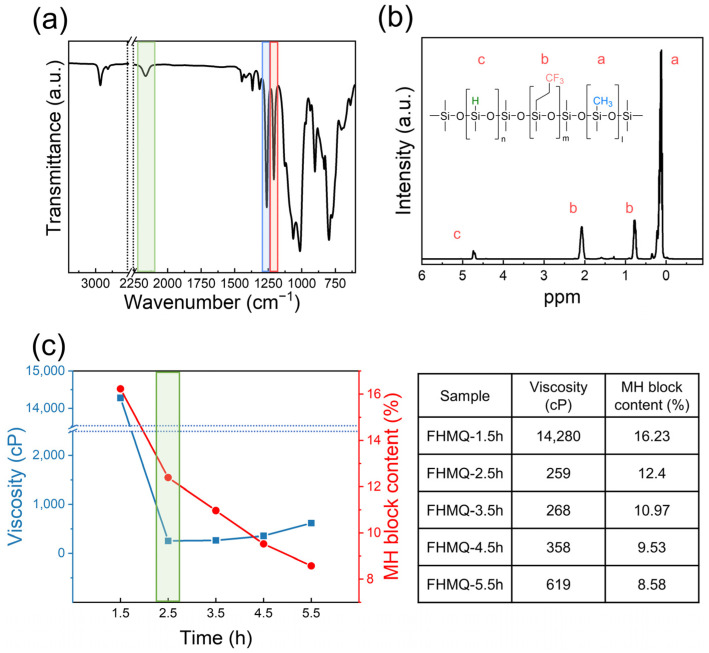
(**a**) FT-IR spectrum of the FHMQ-2.5h; (**b**) ^1^H-NMR spectrum of FHMQ-2.5h, and (**c**) MH-block content and viscosity data of all FHMQs with respect to reaction time.

All the characterizations of the FHMQs are shown in Figure 6. Apart from the characteristic absorption bands of the trifluoropropyl and methyl groups observed in silicone, the absorption band at 2245 cm^−1^ for the Si-H bond was also detected [34]. Furthermore, the characteristic ^1^H-NMR peak of Si–H at 4.7 ppm was clearly observed, as shown in Figure 6b. The correlation between the viscosity and methyl-hydrido block (MH-block) content over the reaction time is shown in Figure 6c. First, the viscosity of the FHMQ-1.5h sample was 14,280 cP, which is much higher than expected. It was assumed that the end-blocker of MM could not be entirely reacted, unlike HD_4_, because the reactivity of linear siloxane was lower than that of cyclosiloxane owing to the low ring strain [35]. Therefore, the long molecular chain of FHMQ and high viscosity at a reaction time of 1.5 h were caused by insufficient chain transfer. Conversely, the viscosities of the rest of the FHMQ samples, with a reaction time of 2.5 to 5.5 h, were below 1000 cP, as expected. Meanwhile, the MH-block content calculated from the peak area in the NMR spectrum decreased with increasing reaction time. This is because when the reaction time is extended, a small amount of water added to the initiator reacts with Si–H to form a Si–OH bond. The modified Si–OH groups condense to form Si–O–Si bonds, resulting in an unexpected increase in the chain length [36,37]. This is the same reason why the viscosity of the samples FHMQ-2.5h to FHMQ-5.5h gradually increased. In this study, FHMQ was targeted with an MH block of more than 10% and a viscosity of less than 1000 cP in consideration of processability. Consequently, FHMQ-2.5h was selected as the crosslinker for the preparation of F-LSR.

### 3.4. Preparation and Characterization of F-LSR

F-LSR was prepared using synthesized FVMQ and FHMQ. A small amount of silica was added to enhance the mechanical properties. Most of the F-LSRs were prepared successfully, but we failed to cure FVMQ-400-f10 under the same conditions, consequently failing to acquire a satisfactory F-LSR-400-f10 sample. The F-LSR-400-f10 sample had many cracks in the sheet, which was too weak to be measured for its mechanical properties. However, the FT-IR spectra of the remaining successfully manufactured F-LSRs were almost identical to those of the FVMQ. In particular, it was confirmed that curing was complete as the Si–H absorption band at 2247 cm^−1^ disappeared (Figure S4).

#### 3.4.1. The Mechanical Properties of F-LSR

**Figure 7:**
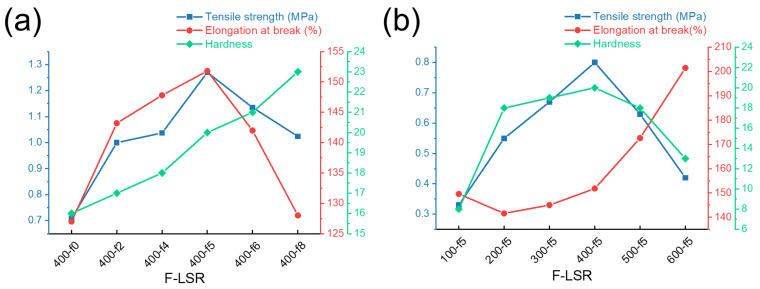
The mechanical properties of F-LSR (**a**) with a different chain-length and (**b**) with different fluorine ratios.

The mechanical properties, such as tensile strength, elongation at break, and hardness, were identified using UTM and a durometer. Figure 7 and Figure S5 and Table 4 show the mechanical properties of all the F-LSRs with a few specific trends. Unfortunately, there are numerous factors affecting the mechanical properties of silicone rubber, such as the crosslinking density, molecular weight, chain length of the main chain, type and amount of functional groups, and silica interaction [38,39,40,41,42,43]. Although it is difficult to directly compare the mechanical properties of the F-LSRs in this study, several important points influencing the mechanical properties were determined from the experimental results. First, the hardness of the F-LSR with different functional groups increased gradually as the MF block increased (Figure 7a). This phenomenon is attributed to the shorter chain length that occurs when the MF block increases in the molecular chain owing to backbiting [44,45]. In the spring model of the cured silicone rubber, shortening the chain length in silicone can increase the tensile strength and hardness and decrease elongation [29]. However, the growth of the MF-block can prevent chain entanglement owing to the high polarity of the trifluoropropyl groups, resulting in reduced elongation and tensile strength [46]. In addition, the Si–H/Si–Vi ratio related to the crosslinking density from F-LSR-400-f0 to F-LSR-400-f8 decreased from 0.599 to 0.454, which can cause a decrease in the tensile strength and elongation [34]. Due to the simultaneous occurrence of several complex effects, the hardness increased as the MF block increased, and the tensile strength and elongation increased for F-LSR-400-f5 and then decreased rapidly.

Second, when the viscosity increases with the same ratio of the MF block, the elongation generally increases, except for FLSR-100-f5 (Figure 7b). Generally, elongation with identical amounts of functional group was directly proportional to molecular weight [47]. According to the spring model, the tensile strength decreases when the molecular chain length increases. However, it has been reported that as the chain length increases while the vinyl group ratio is maintained, many entanglements occur, resulting in a decrease in Mc and an increase in the crosslinking density of silicone [48,49]. Consequently, the hardness and tensile strength of F-LSR-100-f5 to F-LSR-600-f5 increased to an optimum point (F-LSR-100-f5) and then decreased.

#### 3.4.2. Heat Resistance of F-LSR

**Figure 8:**
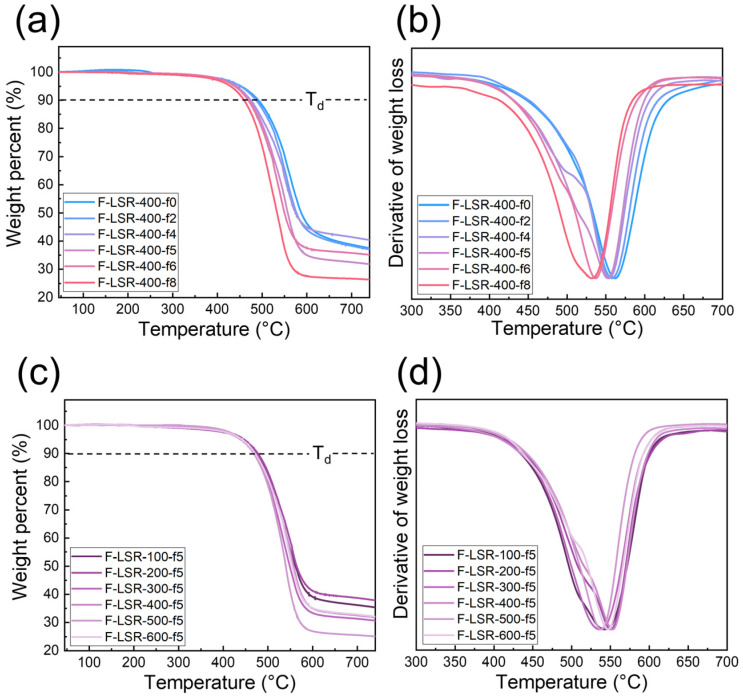
TGA thermogram and derivation of TGA curves of F-LSRs: (**a**,**b**) with different fluorine ratios and (**c**,**d**) with different chain lengths.

The thermal stabilities of FVMQ and F-LSR were measured using TGA at temperatures of 50–700 °C and a heating rate of 10 °C/min under a nitrogen atmosphere (Figure 8 and Table 5). The initial weight loss was less than 5% up to 200 °C, which is ascribed to small amounts of unremoved residue [50]. In order to evaluate the thermal properties, the onset degradation point (T_d_) was set as 10% of the original weight. In addition, it was designated as T_dmax_, where the temperature with the fastest weight-loss rate is shown in Figure 8b. The degradation point of F-LSRs declined gradually from 550 to 460 °C as the number of trifluoropropyl groups increased from F-LSR-400-f0 to F-LSR-400-f8. Moreover, T_dmax_ shifted toward a lower temperature upon increasing the trifluoropropyl group content in F-LSR. Due to the increased polarity derived from the trifluoropropyl groups, the molecular chain of fluorosilicone, with high trifluoropropyl groups, is readily depolymerized at high temperatures to revert to cyclic siloxane [11,51]. In addition, a similar trend for the thermal degradation of FVMQ was confirmed in the TGA and DTGA graphs, shown in Figure S6. The TGA and DTGA curves of F-LSR with different molecular chain lengths are shown in Figure 8c. The T_d_ for FLSR-100-f5 to FLSR-600-f5 is at about 490 °C, and all T_dmax_ is located in the range of 538–549 °C. Consequently, the thermal properties at high temperatures are governed by the fluorine content rather than the molecular chain length.

#### 3.4.3. Thermal Properties and Elastic Retention of F-LSR at Low Temperature

**Figure 9:**
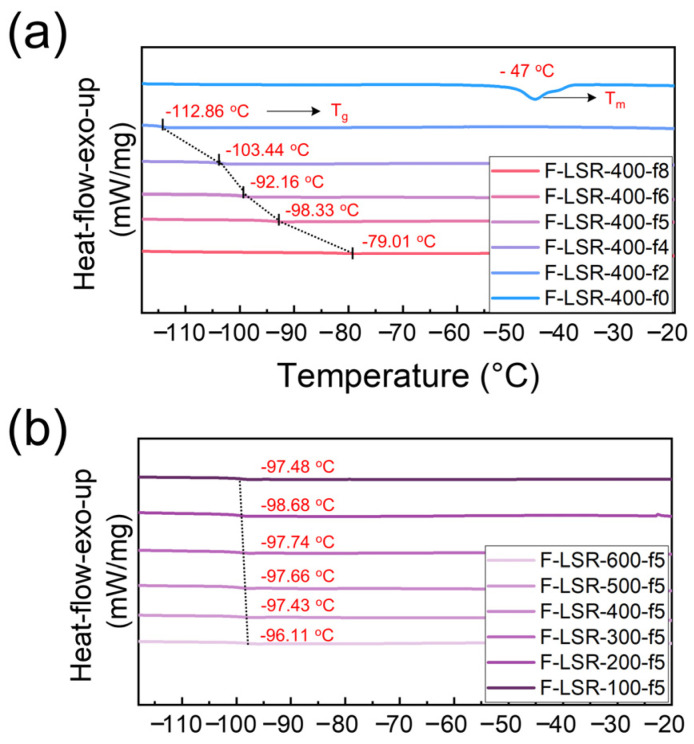
DSC curves of F-LSRs: (**a**) with different fluorine ratio and (**b**) with different chain-length.

DSC analysis of F-LSR and FVMQ was conducted to examine their thermal properties at low temperatures (Figure 9 and Figure S5). In Figure 9a, a peak representing the melting point was found at −47 °C in the DSC curve of FLSR-400-F0. Melting bimodal peaks appeared at −45.7 and −34.8 °C in the DSC curve of A, and the crystal region caused by “cold-crystallization” can be identified at −97.3 °C. A slight difference in the DSC curves of FLSR-400-f0 and FVMQ-400-f0 was induced by a small amount of FHMQ as a crosslinker for F-LSR. The melting points of the other F-LSRs, apart from F-LSR-400-f0, were not observed in the DSC curves. No crystalline regions in other F-LSRs exist because the trifluoropropyl group from F-D_3_ breaks the symmetry and regularity of the polysiloxane monomer [11]. In addition, the high polarity of the trifluoropropyl group prevents the stacking of molecular chains by repelling each other [11,47,52]. Therefore, the crystallization of F-LSR with a high fluorine ratio is challenging because of the hindrance from the trifluoropropyl group. Interestingly, the T_g_ point in DSC appeared at −112.86 °C when FH-block was 20%, and as the MF-block content increased to 40, 50, and 60%, the T_g_ point moved to −103.44, −92.16, and −98.33 °C, respectively. Finally, when the MF-block content is 80%, T_g_ is formed at −79.01 °C, which is approximately 45 °C higher than that of PDMS. Moreover, if the T_g_ of F-LSR with 100% MF-block is inferred from the DSC data of FVMQ-400-f100, it is likely to be formed at around 65 °C, which was also confirmed in the previous literature [33]. In other words, as the content of the trifluoropropyl group in the chain increases, it can be seen that the T_g_ significantly shifts toward the higher temperature. This phenomenon is attributed to the introduction of the trifluoropropyl group for two reasons: (1) the trifluoropropyl group increases the rigidity of the molecular chain owing to its high polarity and steric hindrance due to its bulky size [6], and (2) an increase in the asymmetry of the molecular chain resulting from the trifluoropropyl and methyl groups might alter T_g_ [11]. In contrast, the T_g_ of F-LSR with the same fluorine ratio did not change dramatically, even if the molecular length was significantly changed. It is negligible to shift T_g_ because of the increasing entanglements derived from the long molecular chain.

**Figure 10:**
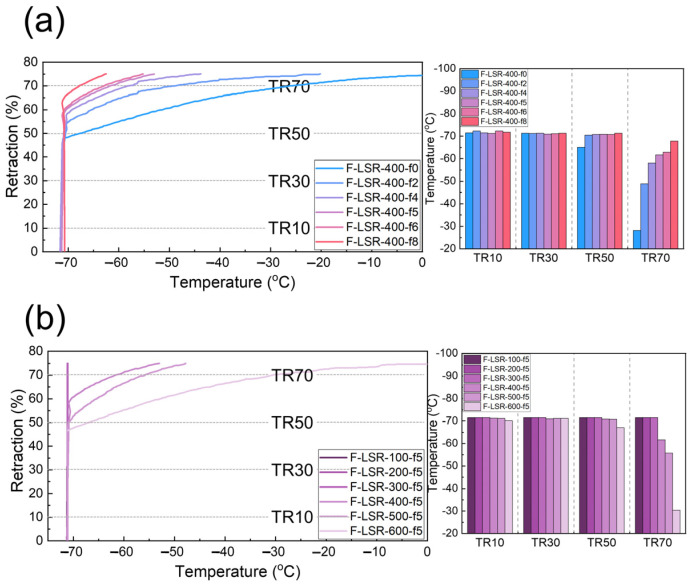
Retraction graphs and analyzing the TR temperature for F-LSRs: (**a**) with different fluorine ratios and (**b**) with different chain lengths.

The TR test was performed to confirm the elasticity retention ability of F-LSR at low temperatures. The TR test started at −75 °C and ended at a temperature restored to 75% of its strained length. In addition, the temperatures at the times of recovery of 10, 30, 50, and 70% from the initial strained length were defined as TR10, TR30, TR50, and TR70, respectively [53]. The extent of retraction of all the F-LSRs is illustrated in Figure 10. Silicone has an excellent capacity to maintain elasticity at low temperatures because T_g_ and T_m_ are formed at lower temperatures compared to other carbon-based polymers owing to the Si–O bond with a long bond length and easily changeable bond angle. Thus, the TR10 and TR30 of all F-LSRs were placed below the test start temperature of 70 °C. The TR50 and TR70 of the F-LSR-400-f0 prepared with FVMQ 400-f0 (as the main chain without fluorine groups) were confirmed at −65.1 and −28.2 °C, respectively. In addition, as the fluorine group in the F-LSR increases, the TR50 and TR70 of the F-LSR are located at lower temperatures. In other words, as the fluorine group inside the F-LSR increases, the better the elastic retention is at low temperatures. Interestingly, although the T_g_ of the F-LSR with a high fluorine ratio was higher than that of the F-LSR with a low fluorine ratio, the elastic retention at low temperatures improved as the fluorine group increased. This indicates that the amount of energy change at T_g_ is so small that it does not significantly affect the elastic retention despite the high T_g_ induced by the trifluoropropyl group. Antithetically, the high polarity of the trifluoropropyl group blocks the crystallized molecular chain, which makes F-LSR amorphous and flexible at low temperatures [6]. The retraction curves of the F-LSR with different chain lengths are shown in Figure 10b. For F-LSR-100-f5 to F-LSR-300-f5, we failed to find the TR70 because all of the strained samples were recovered below 70 °C, despite the presence of 50% MF-block. The TR 70 from F-LSR-400-f5 to F-LSR-600-f5 was located at −61.7, −55.8, and −30.4 °C, respectively, and the low-temperature elastic retention decreased as the molecular length increased. This result suggests that the longer the molecule, the more entanglement between the molecular chains occurs, which makes it easier to crystallize. As a result, it was confirmed that both the MF block ratio and the chain length of the F-LSR significantly affect the TR test results. In the low-temperature embrittlement test, only the F-LSR-400-f0 sample broke at −67.5 °C, while the others did not break at −70 °C. Therefore, the embrittlement temperature could not be determined. Because the hardness of all the specimens was insufficient to proceed with the embrittlement test, additional tests on specimens with a higher hardness were required.

## 4. Conclusions

F-LSRs with various fluorine content and chain lengths were prepared by crosslinking FVMQ and FHMQ. In the synthesis of FVMQ as the main chain and FHMQ as a crosslinker, a TMAS catalyst with superior initiating performance and facile neutralization was used as an initiator to copolymerize several cyclosiloxane monomers. The synthesis of FVMQ was optimized using TMAS, and the FVMQs with diverse molecular weights and fluorine content in their chains were synthesized by changing the ratio of monomers to end-blockers.

(1) All FVMQs were synthesized as random copolymers, and the ratios of the functional groups were almost identical to their theoretical values, with errors of less than 2% determined by ^1^H- and ^29^Si-NMR. Furthermore, GPC confirmed that the molecular weight of the FVMQs could be controlled by changing the loading of the end-blockers. The synthesized FHMQ was also successfully optimized to achieve an MH block content of more than 10% and a viscosity of less than 1000 cP;
(2) The mechanical properties of all F-LSRs were confirmed: (1) the hardness of F-LSR increased as the proportion of fluorine in the chain increased; (2) at a constant fluorine ratio, elongation was proportional to the molecular length, and (3) the tensile strength was the greatest in F-LSR-400-f5;
(3) In TGA, it was confirmed that the thermal stability of the synthesized F-LSR at high temperatures decreased as the fluorine group increased. Conversely, elastic retention and brittleness at low temperatures improved with a high ratio of fluorine groups and long molecular chain lengths.

We expect that this study will provide an excellent guideline for preparing F-LSRs by AROP with a QA initiator, thereby expanding the application of F-LSRs with improved operating conditions.

## Figures and Tables

**Table 1:** Compositions of reactants for the preparation of FVMQs.

Sample	Recipe						Variable
	Monomer Ratio (mol%)				Initiator (wt%)	Time (h)	
	D_4_	F-D_3_	Vi-D_4_	ViVi			
FVMQ-100-f5	49	49	2	1	0.3	5	Viscosity & molecular weight
FVMQ-200-f5	98	98	4	1			
FVMQ-300-f5	147	147	6	1			
FVMQ-400-f5	196	196	8	1			
FVMQ-500-f5	245	245	10	1			
FVMQ-600-f5	294	294	12	1			
FVMQ-400-f0	392	0	8	1	0.3	5	MF-block content
FVMQ-400-f2	313.6	78.4	8	1			
FVMQ-400-f4	235.2	156.8	8	1			
FVMQ-400-f5	196	196	8	1			
FVMQ-400-f6	156.8	235.2	8	1			
FVMQ-400-f8	78.4	313.6	8	1			
FVMQ-400-f10		392	8	1			

**Table 2:** Viscosities and molecular weights of FVMQs obtained through the viscometer and GPC analysis.

Sample	Viscosity (cP)	M_n_ (g/mol)	M_w_ (g/mol)	Ð
FVMQ-100-f5	2,440	32,078	37,810	1.17
FVMQ-200-f5	7,568	55,102	59,278	1.08
FVMQ-300-f5	25,400	46,564	68,608	1.47
FVMQ-400-f5	59,760	55,228	72,089	1.31
FVMQ-500-f5	78,800	76,439	90,356	1.18
FVMQ-600-f5	103,400	85,592	108,909	1.27
FVMQ-400-f0	47,120	76,121	89,424	1.08
FVMQ-400-f2	47,040	75,614	80,934	1.07
FVMQ-400-f4	54,000	57,738	74,153	1.28
FVMQ-400-f5	59,760	55,228	72,089	1.31
FVMQ-400-f6	33,700	48,098	70,180	1.50
FVMQ-400-f8	9,840	32,307	48,904	1.51
FVMQ-400-f10	670	25,758	31,009	1.20

**Table 3:** Theoretical functional groups molar ratio and empirical molar ratio in FVMQs acquired from ^1^H-NMR analysis.

Sample	Theoretical Block Ratio			Empirical Block Ratio		
	DM-Block	MF-Block	MV-Block	DM-Block	MF-Block	MV-Block
FVMQ-100-f5	55.52	41.64	2.83	55.25	42.12	2.63
FVMQ-200-f5	55.68	41.76	2.56	55.84	41.62	2.54
FVMQ-300-f5	55.73	41.80	2.46	55.78	41.70	2.52
FVMQ-400-f5	55.76	41.82	2.42	55.78	41.80	2.42
FVMQ-500-f5	55.78	41.83	2.39	55.36	42.32	2.35
FVMQ-600-f5	55.79	41.84	2.37	55.85	41.9	2.25
FVMQ-400-f0	97.88		2.12	97.93		2.07
FVMQ-400-f2	82.33	15.44	2.23	82.75	15.06	2.19
FVMQ-400-f4	65.1	32.55	2.35	64.25	33.32	2.43
FVMQ-400-f5	55.76	41.82	2.42	55.78	41.7	2.52
FVMQ-400-f6	45.89	51.62	2.49	45.57	51.81	2.62
FVMQ-400-f8	24.34	73.02	2.64	24.89	72.38	2.73
FVMQ-400-f10		97.19	2.81		97.21	2.79

**Table 4:** Mechanical property data of F-LSRs, and information on the Si-H/Si-Vi ratios calculated from the ^1^H-NMR spectrum.

Sample	Tensile Strength (MPa)	Elongation at Break (%)	Hardness	Si-H/Si-Vi
F-LSR-400-f0	0.708	127	16	0.599
F-LSR-400-f2	1	143.2	17	0.566
F-LSR-400-f4	1.037	147.8	18	0.510
F-LSR-400-f5	1.27	151.8	20	0.492
F-LSR-400-f6	1.135	142	21	0.473
F-LSR-400-f8	1.024	128	23	0.454
F-LSR-100-f5	0.467	149.6	8	0.471
F-LSR-200-f5	0.83	141.6	18	0.488
F-LSR-300-f5	1.031	145	19	0.492
F-LSR-400-f5	1.27	151.8	20	0.512
F-LSR-500-f5	1.075	172.6	18	0.528
F-LSR-600-f5	0.652	201.5	13	0.551

**Table 5:** Thermal properties of F-LSRs obtained from TGA, DSC, TR-test, and embrittlement testing.

Sample	T_d_	T_dmax_	T_g_	TR10	TR30	TR50	TR70	T_Em_
F-LSR-100-f5	475.85	542.43	−97.48	−71.5	−71.5	−71.5	−71.5	/
F-LSR-200-f5	477.54	550.35	−98.68	−71.5	−71.5	−71.5	−71.5	/
F-LSR-300-f5	471.31	536.45	−97.74	−71.5	−71.5	−71.5	−71.5	/
F-LSR-400-f5	470.56	552.68	−97.66	−71.2	−71.0	−70.9	−61.7	/
F-LSR-500-f5	468.57	537.96	−97.43	−71.1	−71.1	−70.8	−55.8	/
F-LSR-600-f5	471.46	546.74	−96.11	−70.1	−71.1	−67.1	−30.4	/
F-LSR-400-f0	491.14	561.54	−123.76	−71.5	−71.3	−67.3	−26.1	−65.76
F-LSR-400-f2	486.67	557.77	−112.86	−72.3	−71.2	−70.5	−48.9	/
F-LSR-400-f4	474.20	554.21	−103.44	−71.5	−71.3	−70.8	−58.2	/
F-LSR-400-f5	470.56	552.68	−92.16	−71.2	−71.0	−70.9	−61.7	/
F-LSR-400-f6	468.73	536.76	−98.33	−72.3	−71.1	−70.8	−62.9	/
F-LSR-400-f8	459.6	532.02	−79.01	−71.8	−71.3	−71.3	−67.8	/

## Data Availability

Not applicable.

## Supplementary Materials

The following supporting information can be downloaded at: https://www.mdpi.com/article/10.3390/polym14245502/s1, Figure S1: Description of synthetic methods of TMAS, FVMQ, FHMQ, and F-LSR; Figure S2: Testing methods of TR-test (ASTM: D1329–16) and brittleness test at low temperature (ASTM: D746–20); Table S1. Composition of the reactants for optimizing FVMQ synthesis; Figure S3. ^29^Si-NMR spectra of FVMQs: (a) FVMQ-400-f0, (b) FVMQ-400-f5, and (c) FVMQ-400-f10; Table S2. Composition of reactants for optimizing the synthesis of FHMQ; Figure S4. FT-IR spectra of F-LSRs: (a) F-LSR-100-f5−F-LSR-600-f5 and (b) F-LSR-400-f0−FLSR-400-f8; Figure S5. Stress-strain curves of F-LSRs: (a) F-LSR-100-f5−F-LSR-600-f5 and (b) F-LSR-400-f0−FLSR-400-f8; Figure S6. DSC curves of F-LSRs with (a) different fluorine ratios and (b) different chain lengths.
