# Supplementary material for: Optimization and Characterization of the F-LSR Manufacturing Process Using Quaternary Ammonium Silanolate as an Initiator for Synthesizing Fluorosilicone
Source: Polymers (Basel). 2022 Dec 15;14(24):5502. doi: 10.3390/polym14245502 (PMC9784997; doi:10.3390/polym14245502)
Supplement: Supplementary file 1 [file polymers-14-05502-s001.zip › polymers-2076170-supplementary.pdf]

polymers

MDPI

Article

Supporting information

Optimization and Characterization of the F-LSR Manufacturing Process Using Quaternary Ammonium Silanolate as an Initiator for Synthesizing Fluorosilicone

Jae Il So, Chung Soo Lee, Ji Young Jung, Jaewon Lee, Jin Kyu Choi, Sang Eun Shim and Yingjie Qian

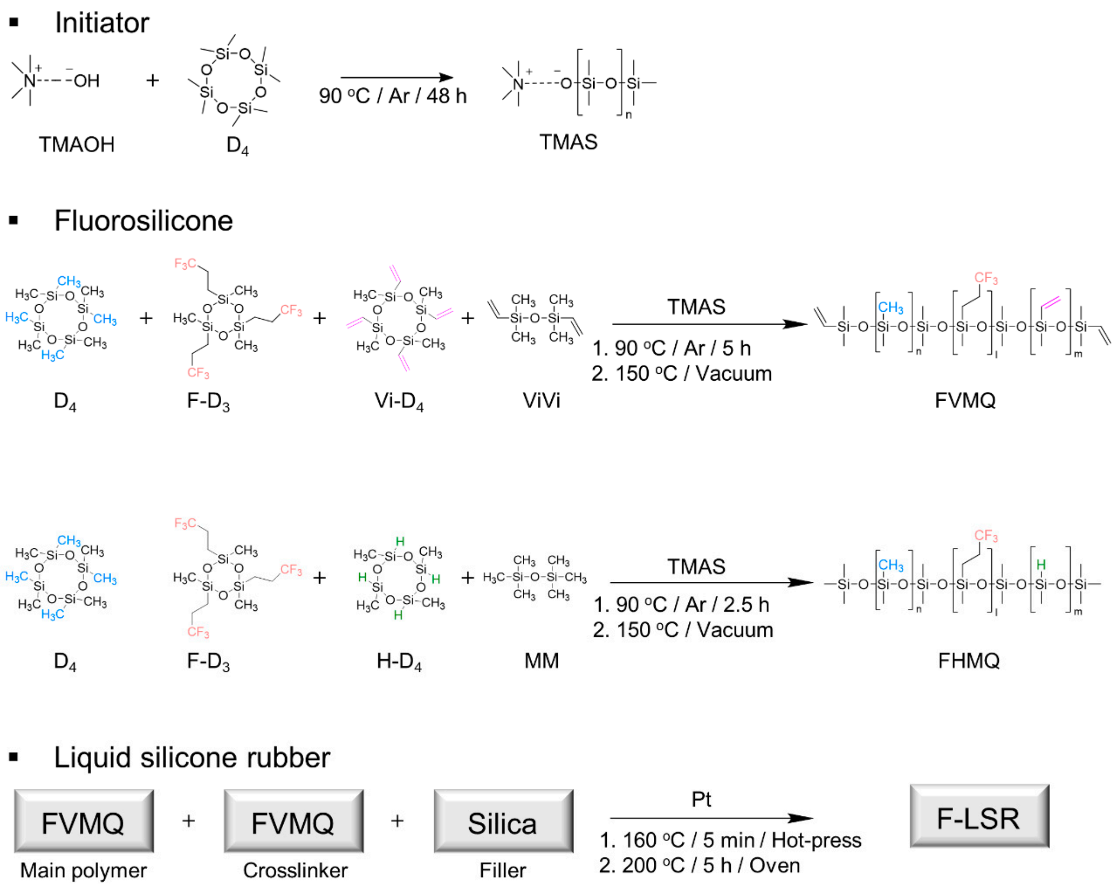

Figure S1. Description of synthetic methods of TMAS, FVMQ, FHMQ, and F-LSR.

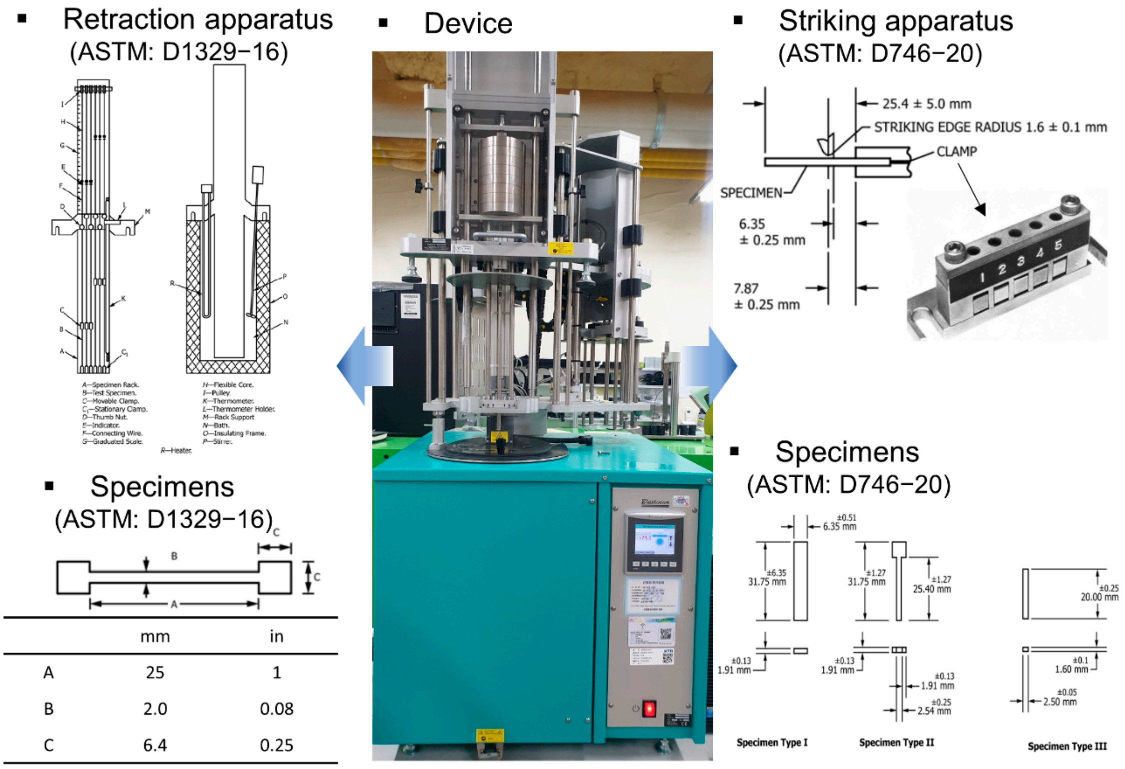

	mm	in
A	25	1
B	2.0	0.08
C	6.4	0.25

Figure S2. Testing methods of TR-test (ASTM: D1329–16) and brittleness test at low temperature (ASTM: D746–20).

Table S1. Composition of the reactants for optimizing FVMQ synthesis.

Sample	Recipe						Variable
	Monomer ratio (mol %)				Initiator (wt%)	Time (h)	
	D_4	F-D_3	Vi-D_4	ViVi			
FVMQ-0.5 h	75	75	0	1	0.2	0.5	Reaction time
FVMQ-1 h						1	
FVMQ-1.5 h						1.5	
FVMQ-2 h						2	
FVMQ-2.5 h						2.5	
FVMQ-3 h						3	
FVMQ-3.5 h						3.5	
FVMQ-4 h						4	
FVMQ-0.1 wt%	75	75	0	1	0.1	3	Amounts of initiator
FVMQ-0.2 wt%					0.2		
FVMQ-0.3 wt%					0.3		
FVMQ-0.5 wt%					0.5		
FVMQ-0.7 wt%					0.7		
FVMQ-1 wt%					1		

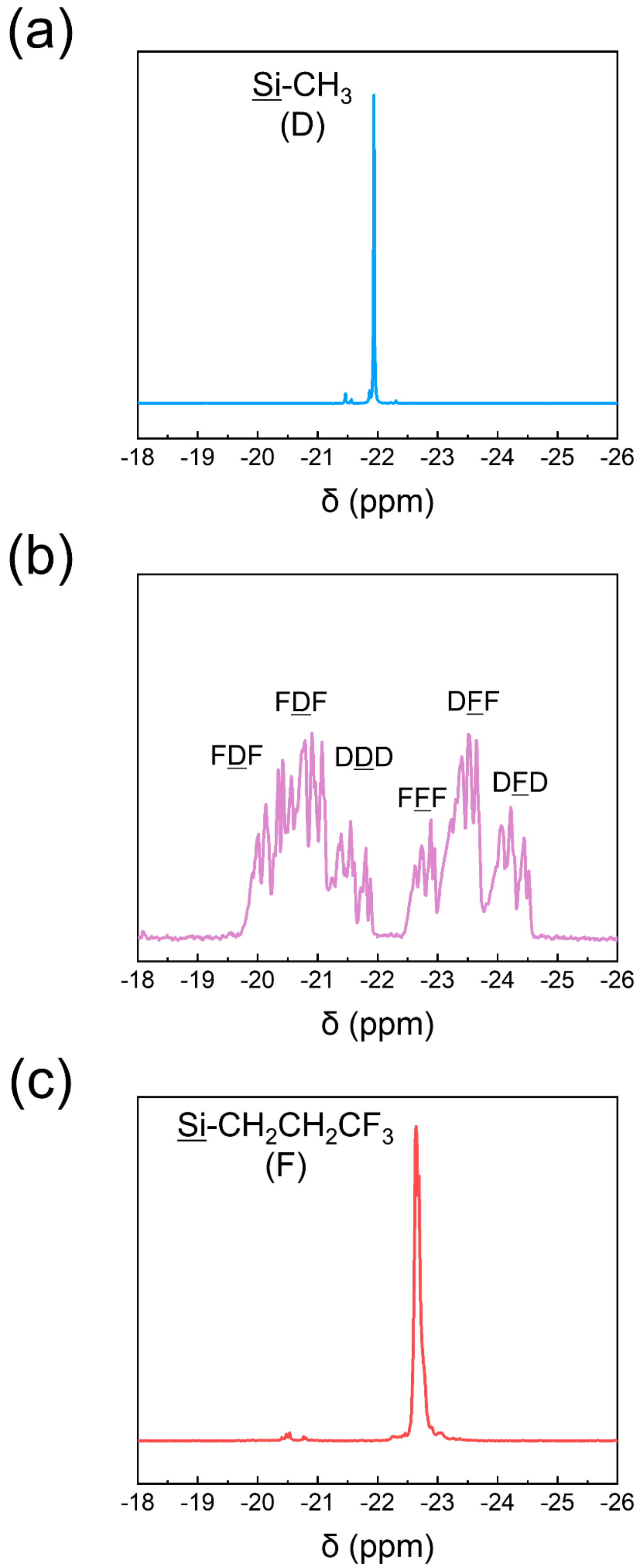

Figure S3. ^{29}Si-NMR spectra of FVMQs: (a) FVMQ-400-f0, (b) FVMQ-400-f5, and (c) FVMQ-400-f10.

Table S2. Composition of reactants for optimizing the synthesis of FHMQ.

**Sample**	**Recipe**						**Variable**
	D_4	F-D_3	H-D_4	MM	Initiator	Time	
FHMQ-1.5 h	1.5	1.5	1	1	0.1 wt%	1.5	Reaction time
FHMQ-2.5 h						2.5	
FHMQ-3.5 h						3.5	
FHMQ-4.5 h						4.5	

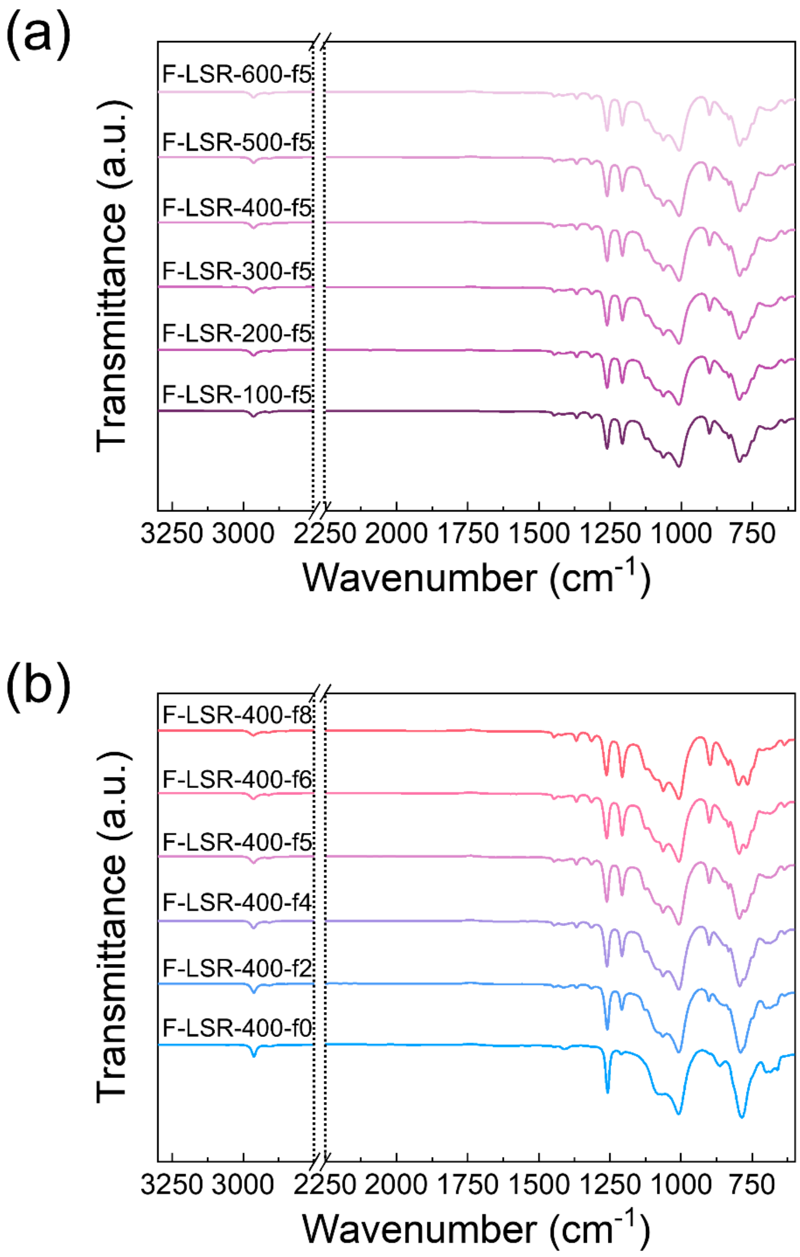

Figure S4. FT-IR spectra of F-LSRs: (a) F-LSR-100-f5−F-LSR-600-f5 and (b) F-LSR-400-f0−FLSR-400-f8.

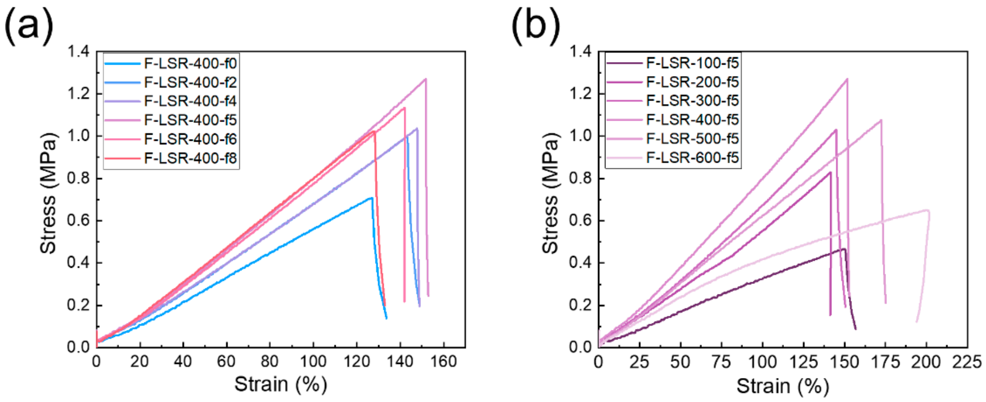

Figure S5. Stress-strain curves of F-LSRs: (a) F-LSR-100-f5−F-LSR-600-f5 and (b) F-LSR-400-f0−FLSR-400-f8.

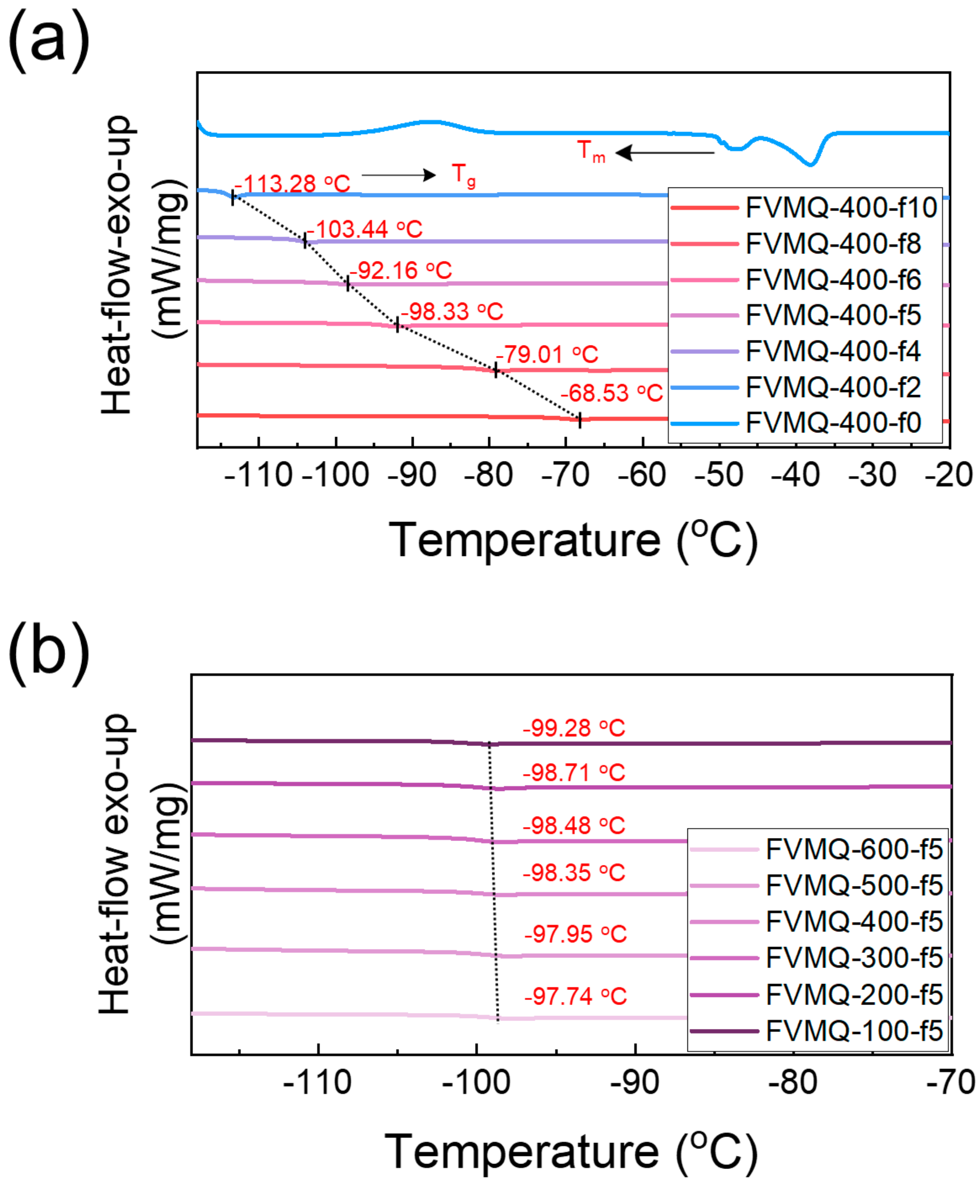

Figure S6. DSC curves of F-LSRs with (a) different fluorine ratios and (b) different chain lengths.
